# Identification and Molecular Characterization of RWP-RK Transcription Factors in Soybean

**DOI:** 10.3390/genes14020369

**Published:** 2023-01-31

**Authors:** Nooral Amin, Naveed Ahmad, Mohamed A. S. Khalifa, Yeyao Du, Ajmal Mandozai, Aimal Nawaz Khattak, Wang Piwu

**Affiliations:** 1 Plant Biotechnology Centre, College of Agronomy, Jilin Agricultural University, Changchun 130118, China; 2 Joint Center for Single Cell Biology, Shanghai Collaborative Innovation Center of Agri-Seeds, School of Agriculture and Biology, Shanghai Jiao Tong University, Shanghai 200240, China; 3 Faculty of Agriculture, Cairo University, Giza 12613, Egypt; 4 Institute of Crop Science Chinese Academy of Agriculture Sciences, Beijing 100000, China

**Keywords:** *RWP-RK* gene family, gene expression, abiotic stresses, *Phytophthora sojae*, nodulation, soybean

## Abstract

The *RWP-RK* is a small family of plant-specific transcription factors that are mainly involved in nitrate starvation responses, gametogenesis, and root nodulation. To date, the molecular mechanisms underpinning nitrate-regulated gene expression in many plant species have been extensively studied. However, the regulation of nodulation-specific *NIN* proteins during nodulation and rhizobial infection under nitrogen starvation in soybean still remain unclear. Here, we investigated the genome-wide identification of *RWP-RK* transcription factors and their essential role in nitrate-inducible and stress-responsive gene expression in soybean. In total, 28 *RWP-RK* genes were identified from the soybean genome, which were unevenly distributed on 20 chromosomes from 5 distinct groups during phylogeny classification. The conserved topology of RWP-RK protein motifs, cis-acting elements, and functional annotation has led to their potential as key regulators during plant growth, development, and diverse stress responses. The RNA-seq data revealed that the up-regulation of *GmRWP-RK* genes in the nodules indicated that these genes might play crucial roles during root nodulation in soybean. Furthermore, qRT-PCR analysis revealed that most GmRWP-RK genes under *Phytophthora sojae* infection and diverse environmental conditions (such as heat, nitrogen, and salt) were significantly induced, thus opening a new window of possibilities into their regulatory roles in adaptation mechanisms that allow soybean to tolerate biotic and abiotic stress. In addition, the dual luciferase assay indicated that *GmRWP-RK1* and *GmRWP-RK2* efficiently bind to the promoters of *GmYUC2*, *GmSPL9*, and *GmNIN*, highlighting their possible involvement in nodule formation. Together, our findings provide novel insights into the functional role of the *RWP-RK* family during defense responses and root nodulation in soybean.

## 1. Introduction

Nitrogen (N) is an important element for plant growth, productivity, and grain quality. It plays a dynamic role in various metabolic activities within the cell, comprising synthesis of a variety of macromolecules such as nucleic acids, proteins, cofactors, chlorophyll, and other molecules involved in signaling and storage mechanisms [1,2]. Generally, only 30% of available soil nitrogen is taken up by plant roots in the form of nitrate (NO^3−^) and ammonium (NH^4+^) ions, amino acids, and other organic molecules. The remaining 70% nitrogen is lost either through leaching into the soil or in gaseous form into the atmosphere, causing environmental pollution. Therefore, efforts are being made to improve crop nitrogen use efficiency (NUE), which will allow high-yielding crops to be grown with a low-nitrogen input without suffering substantial loss of yield [3].

The NUE comprises two major constituents, nitrogen (N) uptake efficiency (NUpE) and nitrogen (N) utilization (assimilation) efficiency (NUtE), although N transport and N remobilization (after assimilation) are two other constituents. Each of these constituents is regulated by several genes [1], including a family of TFs called *RWP-RKs*, which contain a conserved *RWP-RK* motif [4]. These *RWP-RK* genes are ubiquitous in plants and have been categorized in two sub-families including *NLPs* (NIN-like proteins) and RKDs (RWP-RK domain proteins). *NLPs* generally regulate the tissue-specific expression of genes involved in nitrogen use efficiency (NUE) [4,5,6,7], whereas *RKDs* control the expression of genes involved in gametogenesis/embryogenesis [8]. The RWP-RK proteins bind to cis-acting elements in the promoter regions of NUE-related genes (including nitrate reductase (NR/NIA1) and nitrite reductase (NiR1)) and thus regulate gametogenesis and embryogenesis [9]. The DNA-binding RWP-RK domain is shared by both NLPs and RKDs [4,9]. In addition, NLPs also contain an additional octicosapeptide domain called Phox/Bem1 (PB1) that allows interactions with other proteins [5]. The N-terminal regions of NLPs respond to nitrate signals and bind specifically to nitrate responsive elements (NREs) that are found in the promoter regions of nitrate inducible gene loci [5,9,10].

Over the past few years, researchers have explored the genome-wide identification of RWP-RK proteins in various plant species [11,12,13,14,15]. Apart from that, the role of *NLPs* under different NO3−ion concentrations has been demonstrated in Arabidopsis (*Arabidopsis thaliana*), rice (*Oryza sativa*), and tobacco (*Nicotiana tabacum*) [5,9,16]. These studies confirmed that *NLPs* control the expression of nitrate inducible genes including those associated with NUE [5,16,17], whereas *RKDs* take part in N signaling [8]. Nitrate signaling has been shown to post-translationally modify the activity of *NLPs*, leading to the downregulation of *NLPs* and an impairment in the nitrate-inducible expression of numerous genes, which in turn leads to a significant retardation in growth [16,18]. In non-nodulating plants, the frequently studied member of the *RWP-RK* family is *NLP7*, which is involved in the nitrate signal transduction pathway during nitrogen assimilation [10,19]. Studies have also demonstrated that the development of nodules and nitrogen-fixing efficiency could be inhibited by improper regulation of the nitrate-responsive pathway [20]. Similarly, *RWP-RK* TFs have been shown to play partial roles during the transcriptional regulation of stress-related genes. For example, the positive role of the *OsNLP4-OsMADS27* module was described in controlling nitrate-dependent salt tolerance in rice [21]. The *NLP7* protein is also involved during the proper release of BLCs in Arabidopsis, resulting in the defense responses against both biotic and abiotic stresses [22,23]. Although the role of *RWP-RK* TFs during nitrate-specific gene expression has been demonstrated in several plant species, the explicit role of this important class of TFs during nodulation and stress responses in soybean still remain unclear.

Soybean (*Glycine max* L.) is a multipurpose legume crop known for several uses such as feed, oil, and being a nutritional staple [24]. Previously, many reports have presented the genome-wide identification of transcription factor families in soybean, such as *MYB* [25], *MADS-BOX* [26], *NAC* [27], *WKRY* [28], *HD-Zip*, and *bZIP* [29,30], and *ARF* [31]. However, the comprehensive identification and functional characterization of *RWP-RK*-encoding genes in soybean is still lacking today. Due to their significance in plant development and physiology, it is imperative to investigate the discovery and functional analysis of *RWP-RK* genes in the soybean crop. Therefore, this study was designed to unfold the genome-wide identification, structural and functional diversification, conserved topology, and expression profiling of GmRWP-RK-encoding genes in soybean. First, we examined the physicochemical properties, evolutionary relationship, chromosomal mapping, conserved protein motifs, cis-acting elements, and functional annotation of the identified *GmRWP-RK* members. Then, the expression analysis of GmRWP-RK-encoding genes was conducted under *Phytophthora sojae* infection as well as heat, nitrogen, and salt conditions at different time periods. Furthermore, we also carried out the dual luciferase assay of candidate *GmRWP-RK1* and candidate *GmRWP-RK2* during nodule formation. Collectively, the current study provided useful insights into the functional role of *RWP-RK* TFs in promoting NUE efficiency and defense responses against variable stress conditions in soybean.

## 2. Results

### 2.1. Genome-Wide Identification and Physicochemical Properties of Soybean RWP-RK Genes

After manual checking and confirmation using the NCBI conserved domains database, *GmRWP-RK* genes in the soybean genome were identified. Further confirmation was performed with the RWP-RK domain (PF02042) at the Pfam database. The identified members of *GmRWP-RK* genes were also subjected to the hidden Markov model searches (HMM using Arabidopsis *RWP-RK* sequences as input in the BLAST search). After deleting the redundant sequences, a total of 28 *GmRWP-RK* genes were identified from the genome of soybean. The *GmRWP-RK* genes were named sequentially from *GmRWP-RK1* to *GmRWP-RK28* in accordance with their chromosomal locations (Table S1). Furthermore, the different physicochemical characteristics of GmRWP-RK protein revealed that the range of the protein size of *GmRWP-RK*-encoding proteins was between 104–1004 amino acids (*GmRWP-RK11-GmRWP-RK19*). Similarly, the molecular weight of *GmRWP-RK*-encoding proteins were found in the range of 10.08–86.86 kDa (*GmRWP-RK4- GmRWP-RK3*) with an average molecular weight of 42.96 kDa. The ratio of Isoelectric points (pI) was observed as low as 4.79 (*GmRWP-RK15*) and as high as 9.51 (*GmRWP-RK15*). Most of the GmRWP-RK members were detected to be localized in the nucleus, whereas one member (*GmRWP-RK11*) was localized in the mitochondria.

### 2.2. Phylogenetic Relationships of RWP-RKs and Chromosomal Localization

**Figure 1:**
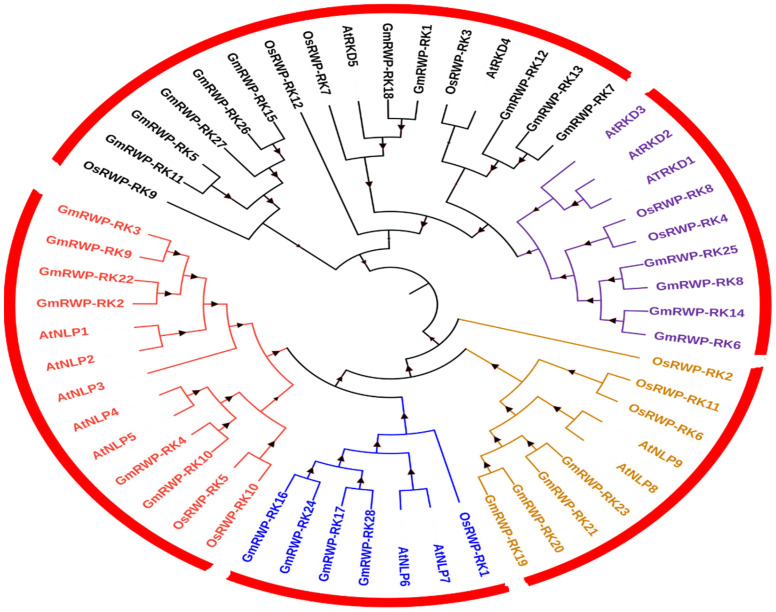
**Phylogenetic analysis of *RWP-RK* proteins from soybean, Arabidopsis, and rice.** A total of 28 RWP-RK members from soybean, 14 members from Arabidopsis, and 12 members from rice were included in the phylogenetic tree using the maximum likelihood method in MEGA version (6.0).

**Figure 2:**
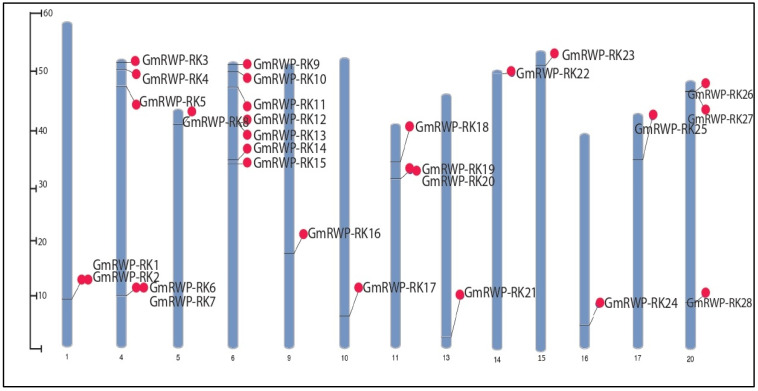
**Chromosomal mapping of *GmRWP-RK* genes in soybean.** The distribution of *GmRWP-RK* genes on 20 different chromosomes are shown with red circles.

To clarify the evolutionary relationships in the GmRWP-RK family, a phylogenetic tree was constructed from 28 soybean GmRWP-RK proteins, 14 Arabidopsis AtRWP-RK proteins, and 12 rice OsRWP-RK proteins using MEGA version (6.0). The putative protein structure showed a configuration of helices, β-pleated sheets, and thin loops (Figure S1), whereas the multiple protein sequence alignment revealed the conservation of specific domains of GmRWP-RK transcription factors (Figure S2). As described in Figure 1, the members of soybean GmRWP-RK proteins were clustered into five distinct groups, designated as Clade-A, Clade-B, Clade-C, Clade-D and Clade-E. The final tree was edited in the iTOL: Interactive Tree Of Life software following the instruction of [32]. The largest clade A included the highest number (10) of GmRWP-RK proteins. The clade B showed the presence of eight members of GmRWP-RK proteins. The remaining three clades C, D, and E were shown to have four members of GmRWP-RK proteins in each clade. Compared with Arabidopsis, most of the GmRWP-RK proteins shared a close relationship with rice, indicating the possibility of functional divergence splits within the gene family over the course of evolution. Similarly, a few members were found in close proximity with Arabidopsis, suggesting these members could have evolved from a similar ancestor exhibiting an identical functional relationship. In addition, the chromosomal localization analysis confirmed that GmRWP-RK genes were randomly distributed on different chromosomes in soybean. The highest number of GmRWP-RK genes were detected on chromosome 6, followed by chromosome 4. The lowest number of GmRWP-RK members were found on chromosome 14 (Figure 2).

### 2.3. Conserved Motif Identification in RWP-RK Proteins

**Figure 3:**
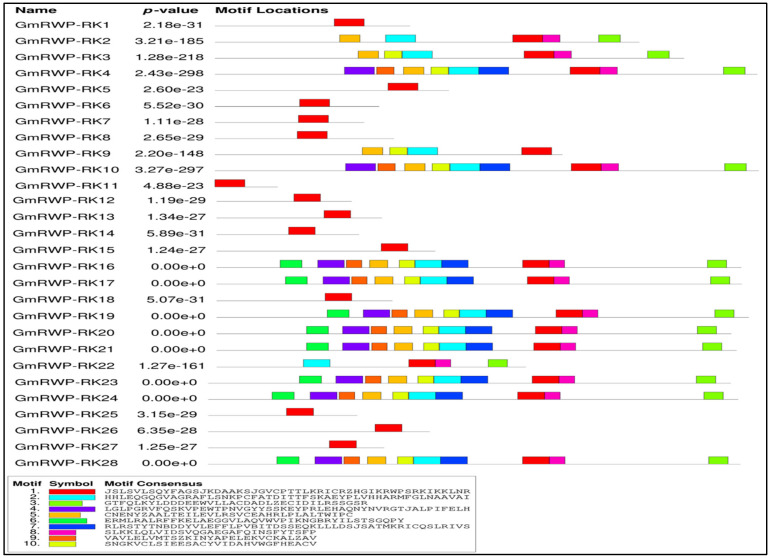
**The distribution and organization of the conserved signature motifs of RWP-RK proteins in soybean.** The protein motifs were analyzed in the MEME server using default parameters. The illustration of different motifs and their sequences are represented by different color boxes.

To identify the conserved motifs’ distribution in *GmRWP-RK* proteins, the MEME online server for motif search was used. A total of 10 protein motifs were identified in *GmRWP-RK* proteins, and these motifs were further annotated with SMART protein-analyzing software (Figure 3). Further analysis of these motifs demonstrated that motif 1 contained the typical domain of *GmRWP-RK* and therefore was found to be well conserved in all members of GmRWP-RK proteins. Importantly, most of the members of GmRWP-RK proteins such as *GmRWP-RK1*, *GmRWP-RK5*, *GmRWP-RK6*, *GmRWP-RK7*, *GmRWP-RK8*, *GmRWP-RK11*, *GmRWP-RK12*, *GmRWP-RK13*, *GmRWP-RK14*, *GmRWP-RK15*, *GmRWP-RK25*, *GmRWP-RK26*, and *GmRWP-RK27* contained the presence of only one protein motif 1, suggesting strong conservation in the C-terminus region. The remaining *GmRWP-RK* proteins showed a variable conservation of 5–10 motifs, which may lead to the functional diversity of these proteins. The conservation of most of the protein motifs was relatively high on the N-terminus, which is in line with other transcription factor-encoding proteins in other plant species. These findings highlighted that *GmRWP-RK* proteins are highly conserved in their functions.

### 2.4. Analysis of the Cis-Elements in RWP-RK Promoters

**Figure 4:**
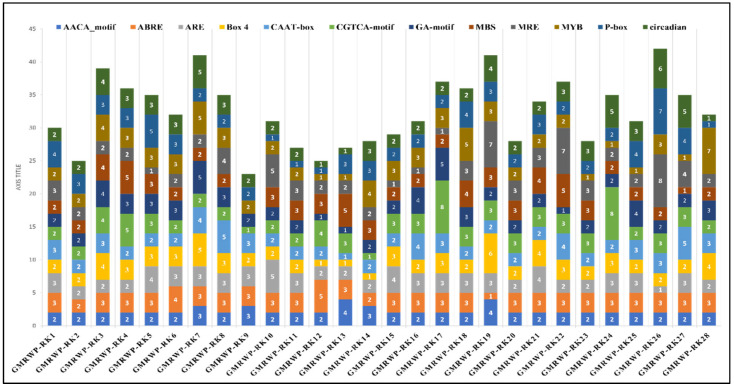
**The distribution of cis-acting elements of *GmRWP-RK* genes in soybean.** The illustration of boxes of different colors shows the occurrence of different cis-regulatory elements within the promoter region of *GmRWP-RK* genes.

To further investigate the regulatory mechanisms and potential role of *GmRWP-RK* genes, the occurrence of cis-elements was detected in the 2-kb promoter region located upstream of the start codon *(ATG)* (Figure 4). The key cis-elements related to growth and development, responses to environmental factors, and phytohormones were mainly detected in the promoter region of all *GmRWP-RK* genes. The meristem-related cis-elements were also identified in the *GmRWP-RK24*, *GmRWP-RK25*, *GmRWP-RK26*, *GmRWP-RK21*, *GmRWP-RK215*, and *GmRWP-RK22* promoters. The stress-related cis-elements of *GmRWP-RK* mainly include circadian, *CGTCA* motif, and *AACA* motif in different *GmRWP-RK* promoters. Similarly, several hormone-related cis-elements, including ABA-responsive element (ABRE), auxin-responsive elements (ARE), and Gibberellin/abscisic acid-responsive element (GA), were all found conserved in *GmRWP-RK* genes. Importantly, the ABA-responsive element of hormonal-related elements occurred (as one to three copies) in all *GmRWP-RK* promoters except the *GmRWP-RK2* promoter. In addition, protein-binding sites including *MYB*, *Box-4*, and *P-Box* and *CAAT-box* were also detected in some *GmRWP-RK* promoters, indicating that the regulation of *GmRWP-RK* genes could be regulated by the induction of other transcription factors.

### 2.5. GO Enrichment Analysis of RWP-RK Genes

**Figure 5:**
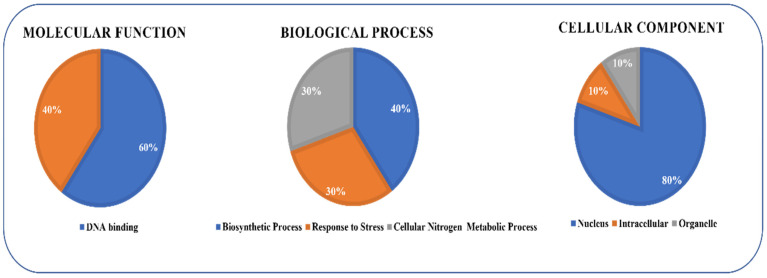
**Gene Ontology (GO) enrichment analysis of the *GmRWP-RK* genes in soybean.** The GO enrichment analysis was represented as three different categories including biological processes (BP), molecular (MF) function, and cellular component (CC).

The gene ontology (GO) of *GmRWP-RK* genes was employed to functionally annotate the selected *GmRWP-RK* genes in soybean. The GO analysis divided the *GmRWP-RK* genes into three main categories including biological processes (BP), molecular (MF) function, and cellular component (CC) (Figure 5). In the molecular function category, 60% of *GmRWP-RK* genes were annotated in the DNA binding processes, whereas 40% of GmRWP-RK genes were found to be enriched in stress responses in biological terms. In the case of the biological process category, 40% of *GmRWP-RK* genes were significantly enriched into biosynthetic pathways, 30% of *GmRWP-RK* genes were annotated in stress responses, whereas the remaining 30% of *GmRWP-RK* genes were significantly enriched into the cellular nitrogen metabolic process. Most of the *GmRWP-RK* genes (80%) were significantly enriched into nucleus, organelle (10%), and intracellular (10%) in the cellular component category. The sub-terms’ classification further highlighted that a larger portion of *GmRWP-RK* genes were significantly enriched in morphogenesis and flower development terms, which are the key biological processes involved in plant development. Similarly, some genes of *GmRWP-RK* were significantly enriched into phytohormonal signaling, suggesting that the expression of these genes could be induced upon different hormone applications under different environmental conditions.

### 2.6. Expression Analysis of RWP-RK Genes Family in Different Tissues

**Figure 6:**
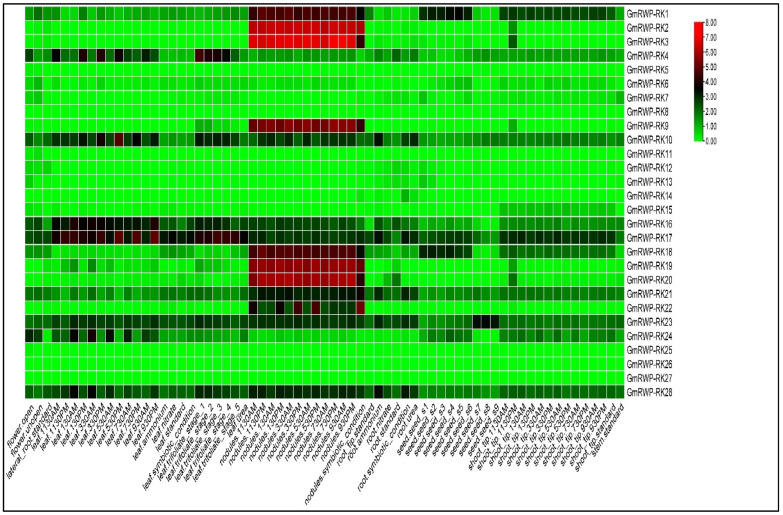
**Expression analysis of *GmRWP-RK* genes in different tissues (seed, nodule, root, leaf, shoots, and flowers) in soybean.** The log2fold values of *GmRWP-RK* genes with the corrected *p*-values were used to construct the heatmap using TBtools software. The red and green colors in the heatmap represent the expression of *GmRWP-RK* genes from bottom to top.

The tissue-specific expression pattern of *GmRWP-RK*-encoding genes was studied using the RNA-seq method to discover the possible function of the *GmRWP-RK* family in soybean. The results showed that the majority of *GmRWP-RK*-encoding genes exhibited low expression across tissues and no expression in many other tissues, suggesting that these genes have mostly redundant roles in soybean development (Figure 6). On the other hand, few members of GmRWP-RK-encoding genes such as *GmRWP-RK1*, *GmRWP-RK2*, *GmRWP-RK3*, *GmRWP-RK9*, *GmRWP-RK18*, *GmRWP-RK19*, and *GmRWP-RK20* showed up-regulation in the nodule tissues at different timepoints. The preferential expression of these GmRWP-RK genes in nodules suggested their potential role in the development of root nodules in soybean. Noticeably, some *GmRWP-RK*-encoding genes, including *GmRWP-RK16*, *GmRWP-RK17*, *GmRWP-RK21*, *GmRWP-RK22* and *GmRWP-RK23*, were slightly expressed in leaf, nodules, seeds, and shoot tissues, indicating the regulatory role of these genes in the vegetative growth of soybean.

### 2.7. Expression Analysis of RWP-RK Genes Family under Phytophthora Sojae

**Figure 7:**
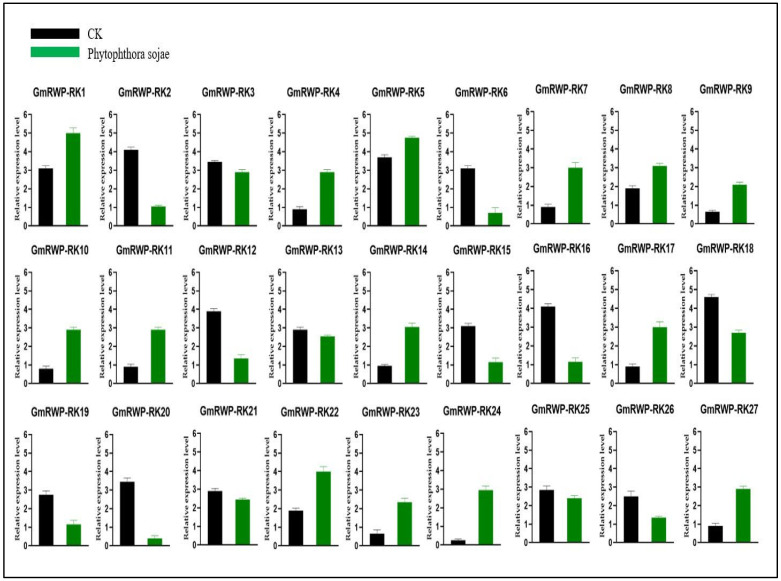
**Relative expression level of *GmRWP-RK* genes in the root tissues of soybean in response to *P. sojae* infection using qRT-PCR analysis.** Error bars show the ±SE from three independent biological replicates.

*Phytophthora sojae* is an oomycete and a soil-borne plant pathogen that causes stem and root rot of soybean. This is a prevalent disease in most soybean growing regions, and a major cause of crop loss. Hence, we carried out the expression analysis of *GmRWP-RK* genes in the root tissue of soybean under *P. sojae* induction. Our results demonstrated that most of the *GmRWP-RK* genes showed significantly increased expression level in response to *P. sojae* treatment. For instance, the expression level of *GmRWP-RK4*, *GmRWP-RK7*, *GmRWP-RK9*, *GmRWP-RK10*, *GmRWP-RK11*, *GmRWP-RK14*, *GmRWP-RK17*, *GmRWP-RK22*, *GmRWP-RK23*, *GmRWP-RK24*, and *GmRWP-RK27* was significantly up-regulated up to three- to four-fold in *P. sojae* treated plants compared to the control plants (Figure 7). Similarly, the expression of *GmRWP-RK1*, *GmRWP-RK5*, and *GmRWP-RK8*, also slightly increased in *P. sojae* treated plants compared to the control plants. On the other hand, the transcriptional regulation of *GmRWP-RK2*, *GmRWP-RK3*, *GmRWP-RK6*, *GmRWP-RK12*, *GmRWP-RK13*, *GmRWP-RK15*, *GmRWP-RK16*, *GmRWP-RK18*, *GmRWP-RK19*, *GmRWP-RK21*, *GmRWP-RK25*, and *GmRWP-RK26* was inhibited and/or reduced in the root tissue treated with *P. sojae* when compared with the control plants (Figure 7). Collectively, the differential expression pattern of resistant *GmRWP-RK* genes and susceptible *GmRWP-RK* genes provided useful clues in understanding the *GmRWP-RK* induced regulatory mechanism of soybean in response to *P. sojae* stress. 

### 2.8. Expression Analysis of RWP-RK Gene Family under Nitrogen Application

**Figure 8:**
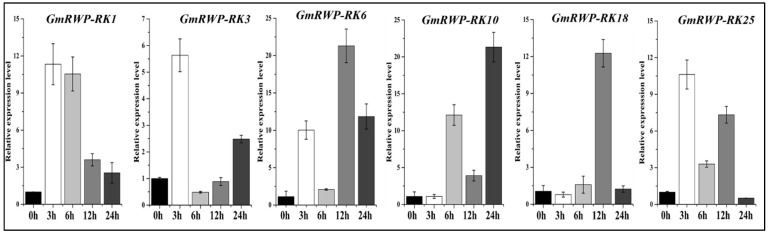
**Relative expression level of *GmRWP-RK* genes under nitrogen application at different time periods using qRT-PCR analysis.** The different colored bars represent different time periods from (0–24 h). Error bars show the ±SE from three independent biological replicates.

Nitrogen is one of the primary nutrients for soybean crops. It is a structural component of chlorophyll molecules and enzymes that helps in the regulation of the physiological processes in soybean. Therefore, an expression analysis of the six selected *GmRWP-RK* genes was conducted in the soybean plants under nitrogen application at different time intervals (0 h, 3 h, 6 h,12 h, and 24 h) using qRT-PCR analysis. Almost all *GmRWP-RK* genes under investigation showed increase expression at the different time points. In particular, the expression of *GmRWP-RK1* and *GmRWP-RK3* genes in soybean seedlings provided with sufficient N application significantly increased at the 3 h time point when compared with control plants (0 h) (Figure 8). Similarly, the expression of *GmRWP-RK6* and *GmRWP-RK18* was up-regulated at the 12 h time point after treatment with N supply. However, the expression level of *GmRWP-RK18* was suppressed at the 3 h time point. Noticeably, the transcript abundance of *GmRWP-RK10* gene reached its maximum at the 24 h time point followed by the 6 h time point compared with the control plants. Importantly, the expression of *GmRWP-RK25* also demonstrated upregulation at the 3 h time point followed by the 12 h time point. However, it was significantly downregulated at the 24 h time point compared to control plants. These findings confirmed the nitrate-specific expression of *GmRWP-RK* genes and could be identified as key regulators of the nitrate-signal transduction pathway in soybean.

### 2.9. Expression Analysis of RWP-RK Genes Family under Heat Stress

**Figure 9:**
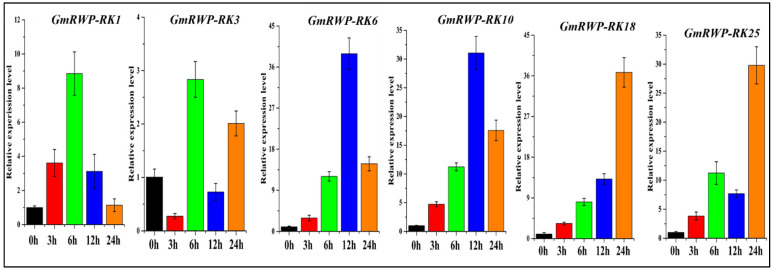
**Relative expression level of *GmRWP-RK* genes in response to heat stress at different time points using qRT-PCR analysis.** The different colored bars represent different time periods from (0–24 h). Error bars show the ±SE from three independent biological replicates.

The relative expression level of six selected *GmRWP-RK* genes was investigated in soybean seedlings subjected to heat stress (42 °C) at different time periods (0 h, 3 h, 6 h, 12 h and 24 h) using qRT-PCR analysis. Noticeably, the results of these *GmRWP-RK* genes showed an increased pattern under heat stress at different time intervals when compared with the untreated plants (Figure 9). For example, the expression of the *GmRWP-RK1* gene under heat treatment significantly increased at 6 h, followed by 3 h, 12 h, and 24 h, respectively, when compared with 0 h (Figure 9). Similarly, the regulation of *GmRWP-RK3* expression reached its maximum at the 6 h and 24 h time points after treatment with high temperature. However, its expression was suppressed at the 3 h time point. The transcript abundance of both the *GmRWP-RK6* and *GmRWP-RK10* genes showed a similar pattern of increased expression at the 12 h time point after heat treatment. In the same way, the two *GmRWP-RK* genes (*GmRWP-RK18* and *GmRWP-RK25*) also demonstrated a consistent pattern of enhanced expression at the 24 h time point after heat stress (Figure 9). These results provided further evidence that *GmRWP-RK* genes are upregulated in response to heat stress and thus open up new gateways into their regulatory role during the adaptation mechanism that allows soybean to tolerate high-temperature stress.

### 2.10. Expression Analysis of RWP-RK Genes Family under Salt Stress

**Figure 10:**
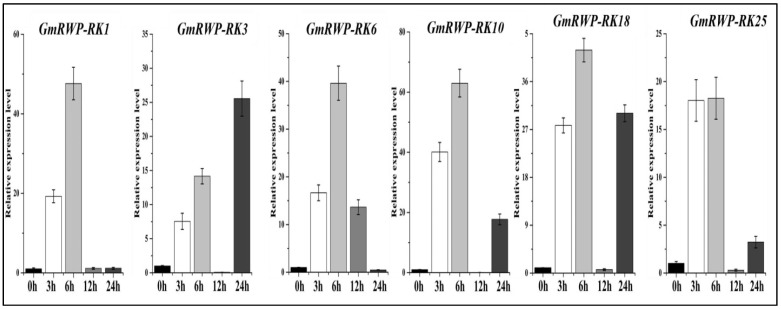
**Relative expression level of *GmRWP-RK* genes in response to salt stress at different time points using qRT-PCR analysis.** The different colored bars represent different time periods from (0–24 h). Error bars show the ±SE from three independent biological replicates.

Salt stress could repress the process of development in crops and adversely influence quality and yield. Here, we investigated the expression profile of the selected *GmRWP- RK* genes in soybean seedlings subjected to salt stress at different time points (0 h, 3 h, 6 h,12 h, and 24 h) using qRT-PCR analysis. The findings from the expression analysis revealed that most of these *GmRWP-RK* genes showed inducible expression level under salt stress at different time intervals compared to control plants (Figure 10). For example, the *GmRWP-RK1* showed high expression at the 6 h time point followed by the 3 h time point; however, its expression was suppressed at the 22 h and 24 h time points. In contrast, the expression level of *GmRWP-RK3* was upregulated under salt stress at the 24 h time point followed by the 6 h and 3 h time points, respectively. However, the *GmRWP-RK3* showed no expression at the 12 h time point. Similarly, the expression level of *GmRWP-RK6* and *GmRWP-RK10* genes significantly increased at the 6 h time point after salt treatment. Importantly, the expression *GmRWP-RK6* plunged at 12 h time point. The transcript abundance of *GmRWP-RK18* and *GmRWP-RK25* reached its maximum under salt conditions at the 6 h time points, followed by the 3 h and 24 h time points, respectively (Figure 10). These findings highlighted important insights into the functional role of these *GmRWP-RK* genes during salt tolerance mechanisms in soybean. 

### 2.11. GmRWP-RK1 Localizations in the Nucleus

**Figure 11:**
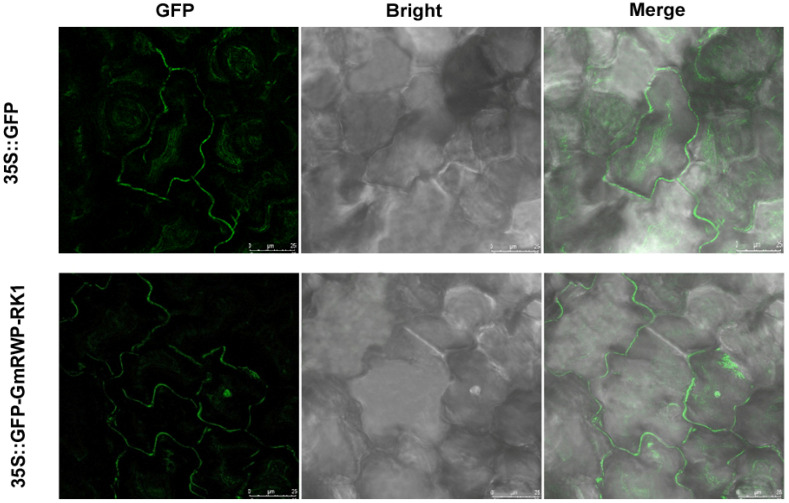
**Subcellular localization of GmRWP-RK1 using the transient expression in *Nicotiana benthamiana*.** The vector containing *35S-GFP* alone was used as a control, whereas 35S-GFP-GmRWP-RK1 represents the recombinant vector containing the GmRWP-RK1 construct. Each row begins with a *GFP* fluorescence, bright field, and merge fluorescence. A confocal laser scanning microscope was used to examine the fluorescence signals (Leica Microsystem CMS, D-68165 Mannheim).

In order to examine the subcellular localization of *GmRWP-RK1*, the online tools UniProt and WoLF PSORT were initially used for the prediction. It was determined that GmRWP-RK1 could be localized and expressed in the nucleus, as demonstrated by the presence of strong nuclear localization signals. To further validate the experimental localization of *GmRWP-RK1*, the transient expression assay of a *GmRWP-RK1-GFP* fusion construct in tobacco leaves was carried out. A laser confocal microscope was used to observe the outcome of the *GFP* fluorescent protein. A green fluorescent signal was mostly observed in the nucleus region when the *GmRWP-RK1-GFP* recombinant vector was compared to the empty vector (Figure 11). This supported the hypothesis that *GmRWP-RK1*-encoded proteins are predominantly expressed in the nucleus (Figure 11). Hence, *GmRWP-RK1* might play an important role in the regulation of important biological and cellular processes crucial to plant growth and development as well in stress responses.

### 2.12. GmRWP-RK1 and GmRWP-RK2 Promote the Transcription of Nodule Specific Genes

**Figure 12:**
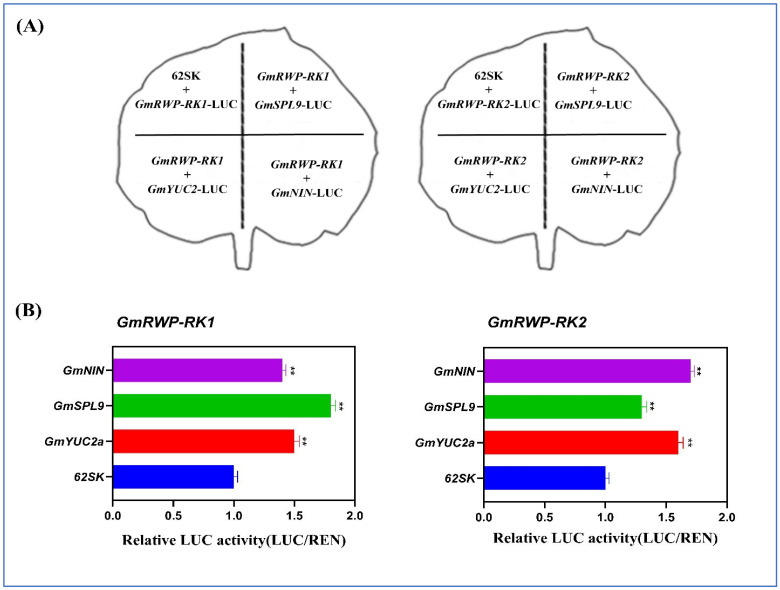
Split-luciferase complementation assay demonstrating the active interaction of *GmRWP-RK1* and *GmRWP-RK2* with *GmYUC2*, *GmSPL9*, and *GmNIN* in tobacco leaves. (**A**) Demonstrates the graphical representation of LUC/gene, LUC/62SK reporter genes with *GmRWP-RK1* and *GmRWP-RK2* with *GmYUC2*, *GmSPL9*, and *GmNIN* (**B**) Demonstrates the relative luciferase activity of the reporter and effector genes, respectively. Error bars shows the standard deviation between samples and asterisks represents ** significant difference at *p* < 0.01.

We speculate that *GmRWP-RK1* and *GmRWP-RK2* are the positive regulators of nodule formation. For this purpose, a dual-luciferase reporter system was established with the *Renilla luciferase* gene under the control of the *GmYUC2*, *GmSPL9*, and *GmNIN* promoters as the reporters, and *GmRWP-RK1* and *GmRWP-RK2* as the effectors. The luciferase signal strength and *RLuc/FLuc* activity were higher in samples transformed with *35S::GmRWP-RK1-EGFP*, *35S::GmRWP-RK2-EGFP*, and the *GmSPL9pro*, *GmYUC2pro*, *GmNINpro* promoters than in samples transformed with *35S::EGFP the GmRWP-RK1*, and *GmRWP-RK2* (Figure 12).

## 3. Discussion

### 3.1. Distribution and Features of RWP-RKs in Plants

The A. thaliana genome encompasses five members of the RKD family, namely *RKD1 (At1g18790)*, *RKD2 (At1g74480)*, *RKD3 (At5g66990)*, *RKD4 (At5g53040)*, and *RKD5 (At4g35590)*. The identification and characterization of the *RWP-RK* proteins have increased our understanding of nitrogen response and gametophyte development in many plant species [4,33]. Soybean is a globally important industrial crop, and the characterization and comparative analysis of the *RWP-RK* proteins will increase our understanding of nitrate response and gametophyte development regulation in *G. max*. In the current study, we identified and characterized 28 *RWP-RK* proteins from the soybean genome database. Cis-acting elements in promoter regions are responsible for modulating the gene expression [34,35]. We found different numbers and types of cis-acting elements in *the RWP-RK* promoters, and these may be responsible for different expression levels of the *RWP-RK* genes in different tissues (Figure 6). However, some *RWP-RK* genes that contained many kinds of cis-acting elements in their promoters nonetheless showed extremely low expression levels in all the tested tissues (Figure 4).

### 3.2. RWP-RKs Regulate Soybean Growth and Stress Responses

The expression of orthologous genes in many tissues of soybean showed different expression levels. Legumes, the third-largest crop in the world, may also produce additional de novo meristems on their roots, which results in the development of lateral roots and symbiotic nitrogen-fixing nodules [36]. The peanut genes *Aradu.YRC2R* and *Aradu.T4VLF* from *Arachis duranensis*, *Araip.R44NW* and *Araip.Y4AFN* from *A. ipaensis*, and *Arahy.K1SYDF*, *Arahy.0FWB0U*, *Arahy.62AJ6F*, and *Arahy.LH2L98* from *A. hypogaea* showed close relationships with the nitrate response genes *AtNLP6* and *AtNLP7* [37,38]. However, the expression level of these genes under N-limited conditions was similar to that under normal conditions, suggesting that their gene expression was not regulated by N [38]. In our study, the majority of the *GmRWP-RK* genes displayed low expression levels in nearly all the tissues except nodules. This suggests that *GmRWP-RK* could be involved in the regulation of soybean growth via nodule modulation and nutrient uptake.

The most important nodule-bearing legume crops, such as *Glycine max* and *Proteus vulgaris*, have not been studied in as much detail as *A. thaliana*. *RWP-RKs* play important roles in both nitrate responses and nodule inception, and they interact with each other to coordinate nitrate signaling and nodulation [39]. Therefore, we assume that a comparison of *RWP-RK* expression and homology in *A. thaliana* vs. legume crops *(G. max and P. vulgaris)* would allow gene functional analyses in model organisms to be applied to nodulating crops. Such studies might also facilitate the transfer of the nitrogen-fixing trait into non-nodulating plants to improve NUE [3,40]. Interestingly, *AtRKD1–AtRKD4* are highly expressed in reproductive organs, and *AtRKD5* has pleiotropic effects on phytohormone pathways, highlighting the regulatory importance of *AtRKD* genes in female gametophyte development [41,42]. In our study, all the genes displayed upregulated expression under nitrogen treatment (Figure 9). These results indicate that *GmRWP-RK* might play a critical role in regulating the soybean response to nitrogen application.

### 3.3. The GmRWP-RK Gene Family Binds and Induces the Transcription of Nodule Specific Genes

The discovery of genes related to flower development and flowering transition remains a major focus in plant research. Studies have shown that MADS-box genes belonging to Type I clade are mainly involved in the development of the female gametophyte, embryo, and seed development, while the functions of plant type II MADS-box genes have a specific role in flower organ development, especially in specifying floral organ identity [43,44]. In most plants, different subfamily members often have different expression patterns [45,46]. In our study, the transcript abundance in flower development of type I clade genes was extremely low, while the subfamilies belonging to the type II clade displayed diverse expression patterns, and most of the type II genes were expressed in the reproductive organs, thereby indicating that type II MADS genes might play important roles in flower development in safflower. To better understand the role of the type II clade in the flower development of safflower, we performed a comprehensive investigation of the expression assay of these genes in different flower organs. The ABCDE model is a classic model of plant flower development [47,48]. Our findings indicated that several classes of MADS-box genes such as A, B, C, and E were expressed at varying levels during flower development, signifying their individual roles in floral organ development in safflower. On the other hand, genes with identical expression patterns may work together in floral organ development. These findings were found to be consistent with the previous studies on plant species, including carnation [49] and orchid [50]. A role for auxin in legume nodulation has long been proposed, but the source of auxin has been elusive. Here, we provide genetic evidence for the auxin biosynthesis gene GmYUC2a as having an important contribution to local auxin production that regulates both rhizobial infection and nodule organogenesis in soybean. Rhizobia induce the expression of GmYUC genes (mainly GmYUC2a) in the root to activate auxin biosynthesis, resulting in a local increase in auxin and the onset of auxin signaling. A possible model may be that auxin signaling is integrated with other hormone signaling pathways to reactivate the cell cycle in root hairs and the cortex for infection thread formation, nodule primordium initiation, and nodule development. In our study, GmRWP-RK1 and GmRWP-RK2 bind to the promoter of the GmYUC2 gene and, in the process, induce its mRNA level. It can be suggested that GmRWP-RK1 and GmRWP-RK2 could work upstream of the GmYUC2 gene, thus encouraging nodule formation. Similarly, GmSPL9d is a typical SPL family transcription factor that interacts with other transcription factors in the nucleus to transcriptionally regulate GmNIN, resulting in nodule formation [51,52]. Here, we observed that GmRWP-RK1 and GmRWP-RK2 positively transcribed the GmSPL9. Importantly, we proved that GmRWP-RK1 and GmRWP-RK2 are nuclear proteins that can directly bind to the promoter of GmSPL9 (Figure 12A,B). These findings enhance our understanding of auxin regulation of nodule development in soybean and provide novel insights into the important legume symbiosis.

## 4. Conclusions

In this study, we presented comprehensive molecular insights into the genome-wide investigation and regulatory role of *GmRWP-RK* transcription factor family in the soybean genome. A total of 28 *RWP-RK* genes were found in the soybean genome, which were scattered over 20 chromosomes that clustered into five different families. The upregulation of *GmRWP-RK* genes in the nodules, as determined by *RNA-seq* data, suggested their crucial roles in the root nodulation of soybean. Additionally, qRT-PCR PCR analysis showed that the expression of *GmRWP-RK* genes considerably increased with a *P. sojae* infection and various environmental conditions including heat, nitrogen, and salt, providing significant clues regarding their potential roles in biotic and abiotic stress responses. Moreover, the dual luciferase test also demonstrated that *GmRWP-RK1* and *GmRWP-RK2* bind to the promoters of *GmYUC2*, *GmSPL9*, and *GmNIN*, suggesting the contribution of these genes in nodule formation. These results will pave the way for identifying candidate *GmRWP-RK* genes involved in the defense responses and root nodulation in soybean.

## 5. Materials and Methods

### 5.1. Phylogenetic and Physicochemical Characteristics of the Gm-RWP-RK Proteins

The protein sequences of the GmRWP-RK family were downloaded from the plant TFDB V4.0 database (http://planttfdb.gao-lab.org/) (accessed on 10 November 2022). The candidate protein sequences were screened and identified using the CDD (http://www.ncbi.nlm.nih.gov/Structure/cdd/wrpsb.cgi) (accessed on 10 November 2022) and SMART (https://smart.embl-heidelberg.de/) (accessed on 10 November 2022), and manual elimination was performed to remove redundant sequences and sequences without typical domains. The Gm-RWP-RK family was finally determined by the protein sequence of the conserved domain [35]. The predicted protein structure of GmRWP-RK was generated by SWISS MODEL tool. To investigate the phylogenetic relationships of the GmRWP-RK members among different plants, multiple GmRWP-RK amino acid sequences were aligned via Clustal omega software using the default settings to examine the evolutionary relationships among the sequences. A phylogenetic tree was constructed with MEGA7.0 software with 1000 bootstrap replicates according to the maximum-likelihood (ML) method [53]. The GmRWP-RK protein structural characteristics, isoelectric point (pI), molecular weight (MW), and grand average of hydropathicity (GRAVY) values were determined using the ExPASy ProtParam tool (http://web.expasy.org/protparam/) (accessed on 11 November 2022). The subcellular localization of GmRWP-RK members was predicted using the online tools UniProt (https://www.uniprot.org/) (accessed on 11 November 2022) and WoLF PSORT (https://wolfpsort.hgc.jp/) (accessed on 11 November 2022)” [53,54].

### 5.2. Conserved Protein Motif and Domain Analysis

The conserved protein motifs of the GmRWP-RK family of *Glycine max* were predicted using MEME Suite (http://meme-suite.org/) (accessed on 11 November 2022) with the default settings. The details of the top 10 predicted motifs were obtained from the MEME suite. The conserved domains of the GmRWP-RK family of Glycine max were predicted using NCBI-CDD (http://www.ncbi.nlm.nih.gov/Structure/cdd/wrpsb.cgi) (accessed on 11 November 2022). The distribution of the conserved domain and motif was drawn via the visualization tool in TB tools software version (1.098769) [55].

### 5.3. Promoter Sequence Analysis

In order to analyze promoter sequence analysis of *GmRWP-RK* gene, the 2000 bp upstream sequence of the initiation codon of each *GmRWP-RK* gene was extracted from the corresponding scaffolds (http://www.kazusa.or.jp/lotus/) (accessed on 14 November 2022). Then, the cis-elements in the promoters of each *GmRWP-RK* gene were predicted using the PlantCARE server (http://bioinformatics.psb.ugent.be/webtools/plantcare/html/) (accessed on 14 November 2022). The distribution of the cis-elements in the promoter was drawn via the visualization tool for cis-elements in TBtools (version 1.098769) software [56].

### 5.4. Plant Materials and Stress Conditions

The experiment was carried out in a control environment. Healthy Williams-82 soybean seeds were planted in plastic pots loaded with sterilized soil. Six seeds were planted in each pot. The loaded pots were kept for 28 days for full growth, and then the pots were subjected to biotic and abiotic stresses with three replications. For biotic stress, *P. sojae* (a soil-borne plant pathogen) was used. Soybean plants were inoculated with *P. sojae* (zoospore) suspension by keeping the loaded pots in 470 mL Styrofoam dishware comprising 200-mL of zoospore “1 × 10^4^” suspension to incorporate into the pots’ soil for 24 h. Then, the pots were detached from inoculation, shifted to a greenhouse and examined on a daily basis to maintain good moisture level. After 10–15 days of inoculation, we observed the root growth, and then the expression of *GmRWP*-*RK* genes was investigated. For abiotic stress, heat stress treatment, the healthy soybean plants were subjected to 42 °C, and samples were collected at different time points. For salt stress, each pot was treated with 120 mM Nacl Hoagland solution. After 0 h, 3 h, 6 h, and 12 h of salt treatment, the plants’ materials were collected and instantly dropped down in liquid nitrogen for real-time quantitative PCR analysis to examine the *GmRWP-RK* gene’s family expression.

### 5.5. Quantitative Real-Time PCR Analysis

To study the expression analysis of the selected *GmRWP-RK* genes, the total RNA extraction was performed, and then the first-strand cDNA template was synthesized using the Revert Aid first strand cDNA synthesis kit (Thermo Scientific), following the manufacturer’s protocol. Specific primers (Table S2) were designed for six *RWP-RK* genes, selected due to their high expression in IVT array digital expression analysis. Each primer pair was checked via RT-PCR followed by 1.2% agarose gel electrophoresis to verify the specificity of the amplification products. The RT-PCR reaction mixture was 20 μL, comprising 2 μL of cDNA template, 0.75 μL each of forward and reverse primers, 1X PCR buffer, 0.25 mmol L^−1^ dNTPs, and 0.5 U Ex-Taq. Thermocycling conditions were as follows: 95 °C for 1 min; followed by 35 cycles of 95 °C for 30 s, 59.9–60.2 °C (depending on primer) for 35 s, 72 °C for 80 s; and a final extension at 72 °C for 7 min [57].

### 5.6. ORF Cloning and Subcellular Localization of GmRWP-RK

The investigation of subcellular localization was performed in tobacco leaves with a transient transformation system. The full-length open reading frame_(ORF) (786 bp) of *GmRWP-RK1* was cloned and fused together with a GFP-containing construct to obtain a recombinant vector of *GFP-GmRWP-RK1* (primers in Table S2). After that, the recombinant vector was inserted into the overexpression vector (pVBG 2307) driven by constitutive cauliflower mosaic virus (CaMV) 35S promoter. Further, it was transformed into Agrobacterium tumefaciens strain GV3101 competent cells. The cells were harvested by centrifugation and re-suspended in a solution containing 10 M MES (pH 5.5), 10 mM MgCl_2_, and 200 μM acetosyringone to an optical density (600 nm) of 0.8–1.0. The cells were then infiltrated into four-week-old Nicotiana benthamiana leaves with a needleless syringe. The agroinfiltrated plants were maintained in a growth chamber for 2–3 days. The tobacco epidermal cells were inspected under an OLYMPUS BX63 automated fluorescence microscope (OLYMPUS, Tokyo, Japan) [34].

### 5.7. Dual-Luciferase Assay

The 1.15-kb promoter fragment of *GmYUC2*, *GmSPL9*, and *GmNIN* was cloned into the pCAMBIA0390-DLuc vector to generate dual-luciferase (Renilla luciferase ad firefly luciferase) reporter constructs Pro*GmYUC2::LUC*, Pro*GmSPL9::LUC*, and Pro*GmNIN::LUC*, in which the firefly luciferase gene under the control of a CaMV35S promoter was used as an internal control. The Pro*GmYUC2::LUC*, Pro*GmSPL9::LUC*, and Pro*GmNIN::LUC* reporter constructs and pCAMBIA3301-EGFP recombinant effector construct were infiltrated into N. benthamiana leaves by Agrobacterium-mediated transformation. Three days after injection, the leaves were harvested and ground into a fine powder in liquid nitrogen to quantify for Renilla and Firefly luciferase (RLuc/FLuc) activity analysis using a Dual-Luciferase^®^ Reporter Assay System (Promega, Madison, WI, USA). The image of the luciferase signal was detected with a plant living imaging system (Lumazone Pylon 2048B, Princeton, NJ, USA).

### 5.8. Statistical Analysis

The data were subjected to an analysis of variance, and the means were compared using Student’s *t*-test at the 5% significance level using SPSS 11.5 software (SPSS, Chicago, IL, USA). The analyzed data were expressed as means ± standard deviation (SD) of three replicates for all measured parameters. Further, the data were plotted by GraphPad Prism 9.4.1 (GraphPad Software, Inc., LA Jolla, CA, USA) [58]. 

## Data Availability

Not applicable.

## Supplementary Materials

The following supporting information can be downloaded at: https://www.mdpi.com/article/10.3390/genes14020369/s1. Table S1: The physico-chemical properties of GmRWP-RK family members in Glycine max L; Table S2: List of Total 28 GmRWK-RK genes primers that are used in the in this experiment; Figure S1: A putative protein structure representation of RWP-RK transcription factor; Figure S2: Multiple protein sequence alignment of RWP-RK transcription factors using clustal omega software.
