# Supplementary material for: Identification and Molecular Characterization of RWP-RK Transcription Factors in Soybean
Source: Genes (Basel). 2023 Jan 31;14(2):369. doi: 10.3390/genes14020369 (PMC9956977; doi:10.3390/genes14020369)
Supplement: Supplementary file 1 [file genes-14-00369-s001.zip › genes-2153826-supplementary.pdf]

Supplementary table S1. The physico-chemical properties of *GmRWP-RK* family members in Glycine max L.

Gene name	Locus ID	CDS	Chr.	Start	End	AA	MW	PI	SL
GmRWP-RK1	Glyma.01G159200	981	Gm01	49710855	49716475	326	37.49	6.53	N
GmRWP-RK2	Glyma.02G311000	2133	Gm01	49710855	49716475	710	79.08	5.87	N
GmRWP-RK3	Glyma.04G000600	2358	Gm04	72328	75769	785	86.86	5.51	N
GmRWP-RK4	Glyma.04G017400	2727	Gm04	1352714	1357474	908	10.08	5.71	N
GmRWP-RK5	Glyma.04G054800	1176	Gm04	4431659	4434088	391	44.61	5.46	N
GmRWP-RK6	Glyma.04G169000	825	Gm04	42346861	42348336	274	32.08	5.52	N
GmRWP-RK7	Glyma.04G234600	750	Gm04	42346861	42348336	249	28.55	8.66	N
GmRWP-RK8	Glyma.05G026500	900	Gm05	2291691	2292932	299	34.92	5.01	N
GmRWP-RK9	Glyma.06G000400	1746	Gm06	46421	49689	581	64.47	6.40	N
GmRWP-RK10	Glyma.06G017800	2733	Gm06	1338317	1343018	910	10.14	5.60	N
GmRWP-RK11	Glyma.06G054900	315	Gm06	4148863	4151186	104	11.91	9.51	M
GmRWP-RK12	Glyma.06G129900	765	Gm06	10677896	10679300	254	29.10	6.20	N
GmRWP-RK13	Glyma.06G130000	936	Gm06	10685232	10687098	311	35.55	7.14	N
GmRWP-RK14	Glyma.06G194300	807	Gm06	17317234	17318568	268	31.46	5.74	N
GmRWP-RK15	Glyma.06G198300	1239	Gm06	18038491	18040823	412	46.29	4.79	N
GmRWP-RK16	Glyma.09G137000	2973	Gm09	33895571	33900191	990	10.95	5.94	N
GmRWP-RK17	Glyma.10G234100	2976	Gm10	46304593	46310226	991	10.97	5.53	N
GmRWP-RK18	Glyma.11G085500	996	Gm11	6431566	6436559	331	38.38	8.74	N
GmRWP-RK19	Glyma.11G125500	3015	Gm11	9530122	9536109	1004	11.12	5.80	N
GmRWP-RK20	Glyma.12G050100	2916	Gm11	9530122	9536109	971	10.76	6.00	N
GmRWP-RK21	Glyma.13G346300	2946	Gm13	43682553	43689937	981	10.78	5.81	N
GmRWP-RK22	Glyma.14G001600	1752	Gm14	202374	204618	583	64.87	6.27	N
GmRWP-RK23	Glyma.15G027900	2922	Gm15	2228855	2237078	973	10.73	5.85	N
GmRWP-RK24	Glyma.16G182400	2964	Gm16	34323618	34328331	987	10.93	6.09	N
GmRWP-RK25	Glyma.17G100400	834	Gm17	7911866	7913909	277	32.11	5.22	N
GmRWP-RK26	Glyma.20G016400	1239	Gm20	1484559	1486779	412	46.40	4.76	N
GmRWP-RK27	Glyma.20G016500	984	Gm20	1491662	1493855	327	36.95	5.69	N
GmRWP-RK28	Glyma.20G160200	2976	Gm20	39860747	39866224	991	10.96	5.64	N

Coding Sequence: CDS, Amino Acid: AA, Molecular Weight: MW, Isoelectric Point: PI, Subcellular Location: SL, N: Nuclear, M: Mitochondrial

Supplementary table S2: List of Total 28 *GmRWK-RK* genes primers that are used in the in this experiment.

#	Gene	Primers	Primer Sequence (5` - 3`)
1	G1RWP-RK1	F	ACAGGGGAAGAAAGGCAAGG
		R	CCAGTGACTCAGACTCGTCG
2	G1RWP-RK2	F	GCGCTTCAACCTAAGCGATG
		R	CGCAGGCTAAAACAACCCAC
3	GlRWP-RK3	F	TGTGTCTCGGTGTTGTCGAG
		R	ACGGCTGCAGGTGGAATAAA
4	GlRWP-RK4	F	CAGCTAACGGTGCTGGTAAC
		R	TTAAGCTCTGCCTCGCTACTG
5	GlRWP-RK5	F	GTCCTCGTAATTGGCCTCCT
		R	TGGCTGATCAATCCGATCCTT
6	GlRWP-RK6	F	CAGAGGTGGCCACATAGGAAG
		R	ATCTCAATGGCAGCCCTAGT
7	GlRWP-RK7	F	GGGCTTTGTCTTTGTCTCCG
		R	TGGTGAAATCTCCATTATTTTGTGA
8	GlRWP-RK8	F	AAGCGTGTTTCAAGGCCAAC
		R	TCTCCCGTTGCGTTAACAAT
9	GlRWP-RK9	F	CAAGGGAAAATCCGGGTCCA
		R	ACGTGCAGATCTACGTTCGG
10	GlRWP-RK10	F	TGACAAAGATCCTGCCACGG
		R	TTCGGGTGTTCACCAAGTGT
11	GlRWP-RK11	F	ATGACTCAGTTGTGTGGTGGA
		R	AACACTATTCTCTTCAACTTCAGG
12	G1RWP-RK12	F	GCAGTACGAAGAAGAGGCCA
		R	GTTGAGCTCCCTGCACCTTT
13	G1RWP-RK13	F	GTCTCTGACCGCGATTTTCC
		R	GCGGTGTCCACCAATCAAGT
14	G1RWP-RK14	F	GGCTGCCATTGAGATGTTGA
		R	ACCTCATGCCCACGAGTTTC
15	G1RWP-RK15	F	GGGCGGGTAAGGAACTGAT
		R	TCTTGTGGGGGATCCACGTTA
16	G1RWP-RK16	F	GAAGCAAGGGCACAAAGTCC
		R	GCATGAACCATGAGGAGTAGGA
17	G1RWP-RK17	F	AAGCAAACAGACAGGCTGGA
		R	CCCAACCTCTCGAGTGTACC

18	G1RWP-RK18	F	CACAGCAACAATTTCCAACCATC
		R	TGTCCGGTGACTCAGACTCATA
19	G1RWP-RK19	F	GCCATAAAGCTTGCCATTGGA
		R	TGTTCATCAGCTCCTTCTACCC
20	G1RWP-RK20	F	GCTGGCTGGAGTAATAGCCC
		R	CACTAAATTCAATCCATCAGGTGGT
21	G1RWP-RK21	F	AGTCTGAAGGATGCCGCAAA
		R	CAGAGTCAAGCACCGTCTGT
22	G1RWP-RK22	F	TCTTCTGGACGTTCCTTGGC
		R	AGTCAAAGATGGAGGCGTGG
23	G1RWP-RK23	F	CCTTCTATGGGGGCGTTTGT
		R	CTTCACTACAAGGTGCGGGT
24	G1RWP-RK24	F	GCAAGGACACGAAGTCCTGA
		R	GGATTTCCACTGCCACTTGAG
25	G1RWP-RK25	F	GGCTGGAACGAAGTTGATGC
		R	TTGCTTCTCGATCCCACCAC
26	G1RWP-RK26	F	CCACGCAATATGCCTCGTTG
		R	ACCCTGCTTCAGTCCTCTCT
27	G1RWP-RK27	F	TGGATTGGACTGAAAGGCAGA
		R	CTACGTCCAGTTTGACCGCT
28	G1RWP-RK28	F	CTCCAACCCCATGTCACCTT
		R	TGTGGCAATCAGAGAAGGCA
29	Actin	F	FCACCAGCCCAAGAAGATC
		R	ATGGCGTTATCCACA
30	GFP-CsCLS21	F	GGGGACTCTTGACCATGGATACAGATTGCAATACGGGGC
		R	TTGCTCACCATCAGATCATTACAAGCCGCCGATGGATGG

Figure S1: A putative protein structure representation of RWP-RK transcription factor. In the below figure spirals denotes helices, whereas broad strips represent β-pleated sheets and thin loops were represented by coils.

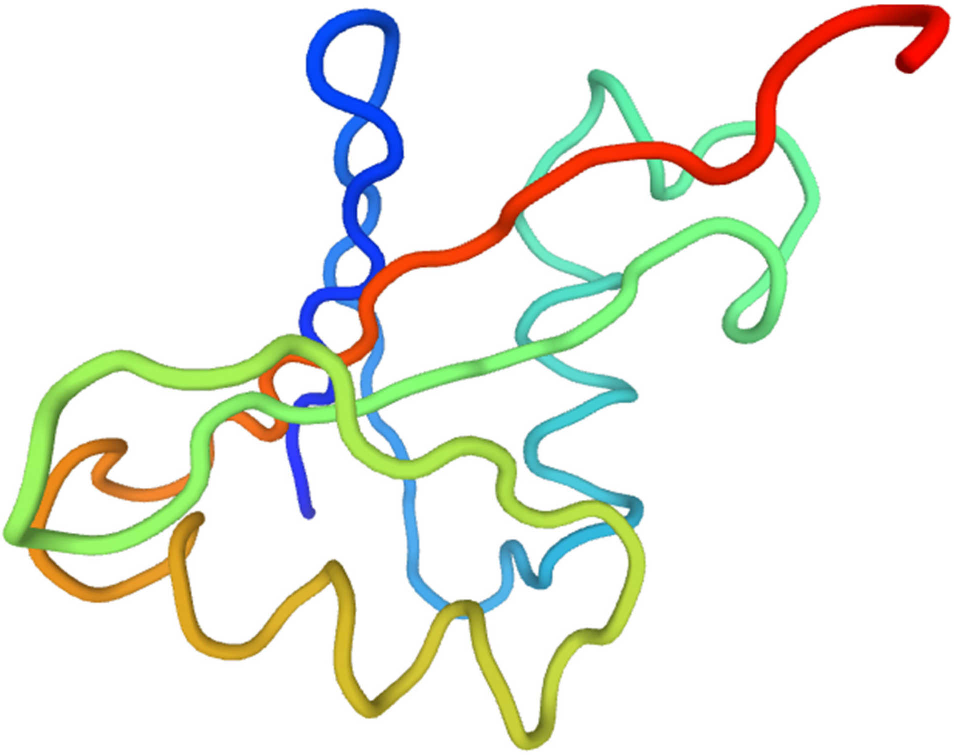

GmRWP-RK-2

Figure S2: Multiple protein sequence alignment of RWP-RK transcription factors using clustal omega software.

```
GmRWP-RK1       -----PVLLVTRNPKLKM-----------IPNL-----LDDL---HVIYKLDRKEE----    127
GmRWP-RK18      -----RLLLVTRNPKLKM-----------IPNL-----LDDL---HMIYKLNRKEE----    132
GmRWP-RK12      -----DLESWNLDFILPP-----------LDED-----LE-E----------LDQKQLKS    92
GmRWP-RK7       -----DLENLDLDFSLPP-----------LGEY-----LEEE----------LDQKPLNI    87
GmRWP-RK13      -----DLENLDLDFSLPS-----------LGEH-----LE-E----------VDQKPSNI    96
GmRWP-RK6       -----DGYHWPYEFPL-------------QDSY-----LDAIPYMKCYYPHDILYES-TL    74
GmRWP-RK14      -----DGYHWPHEFPL-------------QDSY-----LDAIPFMKFYYPHDILYET-TL    75
GmRWP-RK8       -----PSLDWPYDFPI-------------QDYY-----IDAVPLMDYY-PSDPLYETLT-    64
GmRWP-RK25      -----PSLDWPYGFPI-------------QDYY-----LDAVPLMDYY-PSDPLYETLTL    71
GmRWP-RK15      RQINYTNGVRFDKLEIHGTVGVINHAICHVEDKTSNRPPRDTYQIIDFCNKN--------    218
GmRWP-RK26      RQINYTNGVRFDKLEIHGAVGVINHAICLVEDKTSNRPPRDTYQIIDFCNKN--------    218
GmRWP-RK27      RQINYTNGVRFDKLEIHGAVGVINHAICLVEDKTSNRPPRDTYQIIDFCNKN--------    177
GmRWP-RK5       RQTIHTNGTDFERLEIHGRIGLISHIIIQNQSITPGGRSCDNYQMIDFTNGS--------    204
GmRWP-RK11      ------------------------------------------------------------    0
GmRWP-RK21      DISEYPLVHHARKYNLNAAVA-----IRLRSTY----TNDDDYILEFFLPVN--------    492
GmRWP-RK23      DISEYPLVHHARKYNLNAAVA-----IRLRSTY----TNDDDYILEFFLPVN--------    492
GmRWP-RK19      DIGEYPLVHHARKYNLNAAVA-----IRLRSTY----TNDDDYILEFFLPVN--------    530
GmRWP-RK20      DIGEYPLVHHARKYNLNAAVA-----IRLRSTY----TNGDDYILEFFLPVN--------    491
GmRWP-RK17      CKTDYPLVHYALMFGLTSCFT-----ICLRSSH----TGNDDYVLEFFLPPR--------    456
GmRWP-RK28      YKTDYPLVHYALMFGLTSCFA-----ICLRSSH----TGNDDYVLEFFLPPR--------    454
GmRWP-RK16      CKTEYPLVHYALMFGLTSCFA-----VCLQSSH----TGNDDYVLEFFLPPG--------    446
GmRWP-RK24      CKTEYPLVHYALMFHLNSCFA-----VCLQSSH----TGNDDYVLEFFLPPG--------    445
GmRWP-RK4       SKKDYPLSHYARLFGLHAAVA-----IRLRSIY----NSTDDFVLEFFLPVD--------    463
GmRWP-RK10      SKKDYPMSHHARLFGLRAAVA-----IRLRSIY----NSTDDFVLEFFLPVD--------    465
GmRWP-RK3       SKAEYPLAHHANMFGLHAAVA-----IPLLSD-------SADFVLEFFLPKD--------    382
GmRWP-RK9       SKAEYPLAHHANMFGLHAALG-----IPLRSA-------SADFVLEFFLPKD--------    391
GmRWP-RK2       TKAEYPLSHHANIFDLHSAVA-----IPLRTSS----YPHFDFVLEFFLPKH--------    357
GmRWP-RK22      SNAEYPLSHHASIFDLHAAVA-----IPLTTFS-----SSFHFVLEFFLPLD--------    234
```
